# Tumor microenvironment: new era in exosomal circRNA research - a bibliometric analysis

**DOI:** 10.20517/evcna.2024.102

**Published:** 2025-05-19

**Authors:** Hai-Quan Wang, Lu Zhang, Ming-Jie Li, Rong-Quan He, Di-Yuan Qin, Bin Li, Jian-Di Li, Ke-Jun Wu, Shi-De Li, Han He, Zhen-Bo Feng, Gang Chen

**Affiliations:** ^1^Department of Pathology, The First Affiliated Hospital of Guangxi Medical University, Nanning 530021, Guangxi, China.; ^2^Department of Pathology/Forensic Medicine, The First Affiliated Hospital of Guangxi Medical University, Nanning 530021, Guangxi, China.; ^3^Department of Oncology, The First Affiliated Hospital of Guangxi Medical University, Nanning 530021, Guangxi, China.; ^4^Department of Computer Science and Technology, School of Computer and Electronic Information, Guangxi University, Nanning 530004, Guangxi, China.; ^5^Department of Information Management and Information Systems, School of Information and Management, Guangxi Medical University, Nanning 530021, Guangxi, China.; ^6^Department of Clinical Medicine, School of Basic Medical Sciences, Guangxi Medical University, Nanning 530021, Guangxi, China.; ^#^Authors contributed equally.

**Keywords:** Exosomes, circRNAs, TME, visualization, bibliometric

## Abstract

**Aim:** The domain of exosomal circRNAs has seen rapid growth over the past few years, yet a thorough synthesis of the scholarly contributions has been lacking. This study employs bibliometric analysis to uncover the focal points and trends in exosomal circRNA research.

**Methods:** We selected articles on exosomal circRNAs from the Web of Science Core Collection (WoSCC) and deployed VOSviewer for co-authorship and co-occurrence analysis. CiteSpace was tapped to calculate citation bursts for documents and keywords, generating a time-zone map. Additionally, the bibliometrix R package was employed to identify locally cited references and construct thematic maps and trend topics.

**Results:** The exosomal circRNA research field has produced 767 articles over the past decade. China and Fudan University lead in publication counts, ranking first among countries and institutions, respectively. The journal with the highest number of publications and citations is *Molecular Cancer*. Zhang Wei and Li Yan are the top contributors and the most co-cited authors, respectively. Analysis of the results suggests that research related to the tumor microenvironment (TAM) may be a recent research hotspot. Among the latest keywords, terms such as microenvironment, macrophage, mesenchymal stem cells, and ceRNA appear frequently. The keyword with the most recent citation burst is ceRNA. The latest trend topic is the macrophage, and mesenchymal stem cell is identified as an emerging theme.

**Conclusion:** This study elucidates the hotspots and trends in exosomal circRNA research through bibliometric analysis. Themes related to the TAM, especially directions involving macrophages, fibroblasts, and mesenchymal stem cells, will likely open a new chapter in exosomal circRNA research.

## INTRODUCTION

Since the 1980s, a breakthrough in cell biology has been the identification of exosomes, and research on them has been active in various fields in recent years. Exosomes, with a size range of 40-160 nm, are extracellular vesicles that come from endosomes and are generated through the budding process of multivesicular bodies (MVBs)^[1]^. Early theories regarded exosomes as insignificant cellular waste products. However, after years of research, they have been identified as functional carriers containing various molecules such as miRNAs, lncRNAs, circRNAs, proteins, and lipids^[2,3]^. Almost all cells can produce exosomes, which can enter the bloodstream and circulate across the body^[4]^. Exosomes in circulation transport their rich contents to target cells, influencing various physiological and pathological processes like intercellular communication, immune responses, cardiovascular diseases, and cancers^[5-7]^. Currently, research has increasingly focused on the function of exosomes in the tumor microenvironment (TME) and immune responses, as well as their potential for diagnosing and treating diseases^[1,8,9]^.

In the past decade, circRNAs, a new class of non-coding RNAs, have attracted considerable attention. Due to their unique covalently closed-loop structure, circRNAs are resistant to RNase R, providing them with significant stability^[10]^. Although circRNAs were first discovered in RNA viruses as early as 1976, they were once mistakenly considered byproducts of erroneous splicing. This misconception was overturned in 2013 with the proposal of the miRNA sponges concept^[11,12]^. Over 20,000 circRNAs have now been identified, and these circRNAs are widely distributed across various tissues, exhibiting high cell or tissue specificity^[13]^. Certain circRNAs have been identified as being related to cancer progression or metastasis, including liver cancer, lung cancer, and breast cancer.^[14-16]^. They are also linked to non-cancerous diseases, including cardiovascular diseases, diabetes, and autoimmune diseases^[17-19]^. These circRNAs show potential as biomarkers.

In 2015, a study first revealed the close link between exosomes and circRNAs, discovering that exosomes contain abundant and highly stable circRNAs. Through exosomes, these circRNAs enter the bloodstream, and exosomal circRNAs found in serum can help distinguish colorectal cancer patients from healthy individuals^[20]^. Recent studies further suggest that exosomal circRNAs participate in the circulation of plasma, saliva, and urine, and may be related to disease prognosis^[21]^. Exosomal circRNAs derived from cancer cells play roles in tumorigenesis, proliferation, and metastasis, affecting the TME, with some circRNAs enhancing resistance to treatment^[22]^. Furthermore, the clinical potential of exosomal circRNAs has generated considerable interest, as they serve not only as potential biomarkers but also as tools for targeted drug delivery^[23]^.

Research on exosomal circRNAs has been ongoing for several years, but still faces many challenges. Currently, there is no standardized method for extracting and analyzing exosomal circRNAs, making it difficult to replicate study results. Furthermore, circRNAs are present at low abundance, and share sequence overlaps with linear RNAs, making accurate functional assessment challenging^[21,24]^. Additionally, the understanding of basic mechanisms, such as how circRNAs are enriched in exosomes and how they are degraded, is still insufficient^[25]^. Clinically, the diagnostic sensitivity and specificity of most exosomal circRNAs have not yet reached clinical standards, and further screening is required for clinical trials^[26]^. These challenges suggest that research on exosomal circRNAs remains in the early stages. It is crucial to systematically integrate and analyze the current research outcomes and identify future research directions. However, there is currently a lack of systematic analysis of the substantial body of scientific work in this field. Bibliometrics, a mature method of qualitative and quantitative analysis based on literature, can uncover the development process, collaboration networks, research trends, and hotspots of a research field^[27]^. Its use has become widespread in the analysis of multiple disciplines, providing a deeper comprehension of the present state of research. It has been used to study areas such as cancer, central nervous system diseases, and ncRNAs^[28-30]^. As publications on exosomal circRNAs grow and the variety of research topics increases, the numerous dispersed research directions hinder researchers from grasping the full picture of the field. Bibliometric analysis will not only assist in organizing the knowledge system but also promote in-depth research and the clinical application of exosomal circRNAs.

This paper will employ bibliometric analysis tools such as CiteSpace, VOSviewer, and Bibliometrix (an R package) to conduct a comprehensive analysis of exosomal circRNAs, explore the hotspots and future trends in this field, and offer insightful guidance for further research.

## METHODS

### Data collection

**Figure 1:**
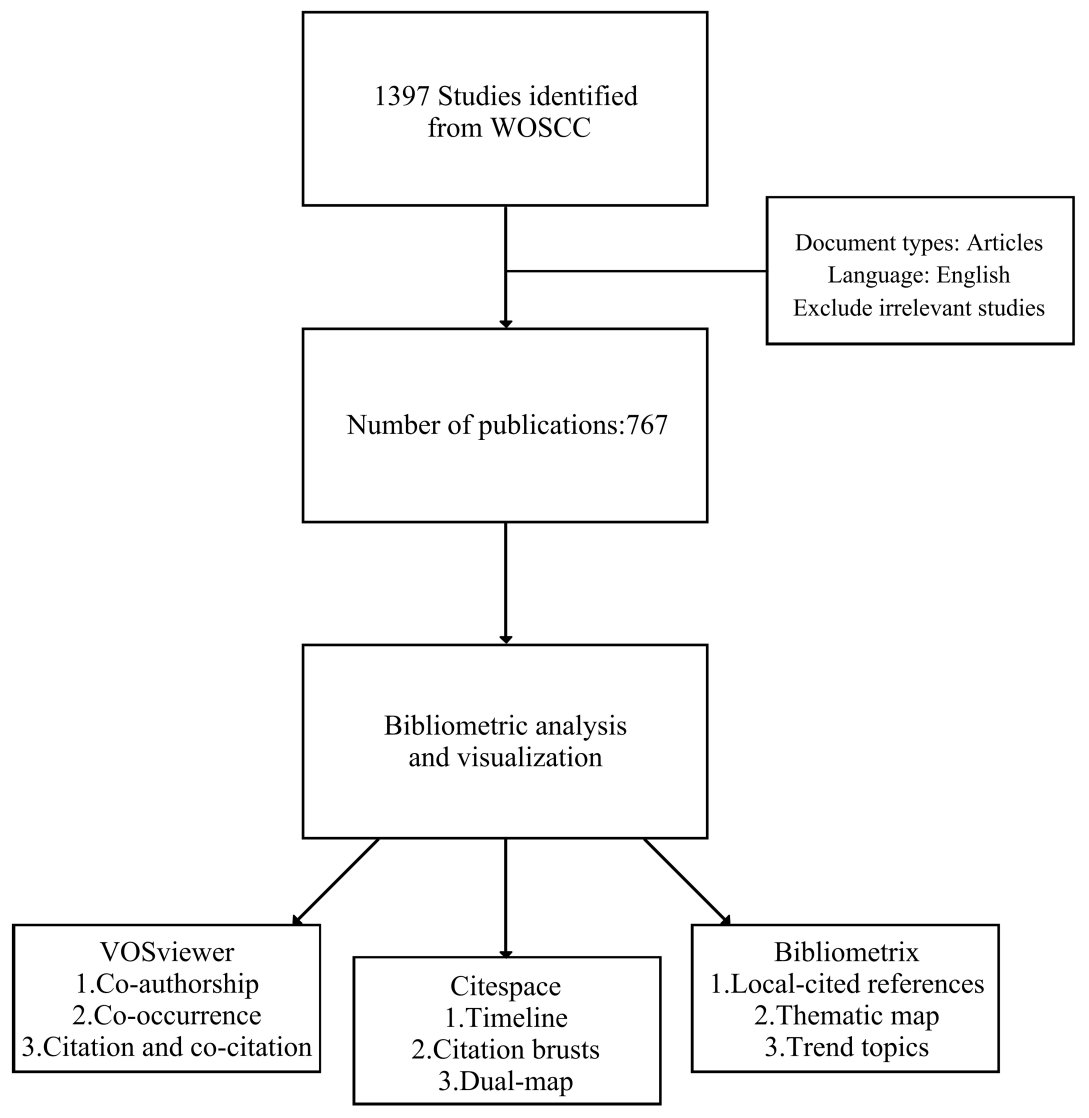
Research process. WoSCC: Web of Science Core Collection.

Tens of thousands of high-quality journals and comprehensive citation records are included in the Web of Science (WoS) database. Compared to Scopus, WoS has a longer index history and more detailed citation analysis. Additionally, unlike PubMed, which focuses on life sciences and medical fields, WoS provides a broader, multidisciplinary perspective^[31,32]^. Furthermore, PubMed does not include references as part of metadata, which is not conducive to bibliometric analysis. Thus, among popular databases, WoS is considered the most suitable for bibliometric studies. This research is based on the Web of Science Core Collection (WoSCC). Literature was retrieved on December 11, 2024, and the screening and export were completed in one day. The search strategy used was: TS = (circRNA^*^ OR “circular RNA^*^” OR “circular noncoding RNA” OR “circular ncRNA” OR “circular nonprotein-coding RNA” OR “circular nonprotein coding RNA”) AND TS = (exosome^*^ OR EVs OR EV OR Extracellular vesicle^*^), resulting in 1,397 articles. Notably, to ensure a comprehensive search, “Extracellular vesicle” was included in the search query, and studies not related to exosomes were excluded during the manual screening phase. The general inclusion criteria were to avoid overlapping topics or low relevance. The detailed criteria are as follows: Inclusion criteria: Only English articles published as “article” type focusing on exosomal circRNAs were included. Exclusion criteria: Articles were excluded if they were classified “article” types (e.g., reviews or conference abstracts), did not have exosomal circRNAs as the main research focus (e.g., studies on extracellular vesicles or non-coding RNAs), or had been retracted. Ultimately, 767 articles were retained. After saving the full record and cited references, the data was exported in Plain Text File format. The research workflow is presented in Figure 1. All data are available in the WoSCC.

### Data analysis

This study used VOSviewer (version 1.6.18), CiteSpace (version 6.4.R1), and the Bibliometrix R package to perform bibliometric analysis on the exported data. VOSviewer is a bibliometric and scientific visualization software tool with clustering algorithms that can effectively analyze knowledge units based on association strength or similarity^[33]^. VOSviewer was employed in this research to carry out most of the data analysis and generate several visual maps, which include networks of countries, institutions, author collaborations, co-cited literature, and keyword co-occurrence. In terms of parameter settings, the counting method was full counting, while the resolution and minimum cluster size were set at 1.0 and 1, respectively. The minimum number of occurrences was adjusted based on the readability of the map.

CiteSpace enables multidimensional and dynamic analysis of disciplines over time. Based on this feature, we used CiteSpace to create temporal zone maps of keywords. We also identified citation bursts and co-citation bursts of literature and keywords. Given the short duration of emerging bursts, we set the minimum duration for keyword burst detection to 1 for greater sensitivity. Additionally, we created a dual-map overlay of journals to reveal relationships between publishing journals and co-cited journals. The configuration parameters were set to a period from January 2015 to December 2024, with yearly time slices to capture the annual dynamic changes in the research field. In CiteSpace, the g-index was used to measure the impact of authors or a group of articles, with the k-value adjusting the threshold to select highly impactful literature. Larger *k*-values result in larger networks. To balance the readability and comprehensiveness of the maps, we set the k-value to 25. The pathfinder method was chosen for pruning, which reduces redundant connections in the network, including pruning sliced networks and pruning the merged network^[34]^. The remaining parameters were kept at their default settings.

R (Version 4.3.2) is a programming language commonly used for statistical computation and graphical expression. In this research, we utilized the Bibliometrix R package for further bibliometric analysis of the collected data, complementing the functionality of VOSviewer and CiteSpace^[35]^. Based on its features, we identified locally cited references from the data. The Walktrap algorithm of Bibliometrix can effectively identify communities in large networks, making it suitable for processing complex co-word networks and revealing the thematic structure within academic literature^[36]^. Therefore, using Walktrap’s clustering algorithm, we generated thematic maps and trend topics for keywords plus to highlight emerging research hotspots.

Additionally, we merged keywords with similar meanings to eliminate duplicate records. Although different software may identify keywords differently, the merging criteria remained consistent: Different forms of the same term. Other expressions of the same term. Highly overlapping research fields.

## RESULTS

### The trend of publication outputs

**Figure 2:**
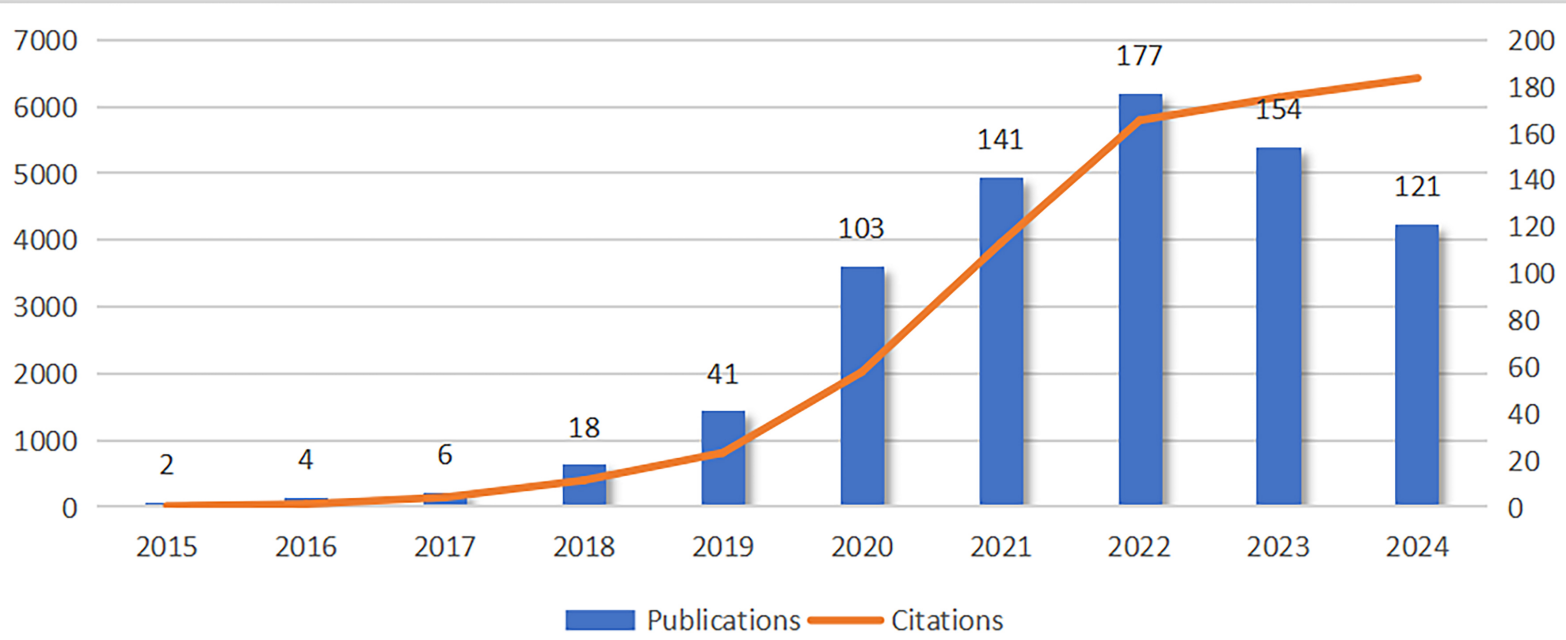
The publication and citation trends of exosomal circRNA research.

After applying our designed search strategy and filtering, we included 767 articles in this analysis. Figure 2 illustrates the trend in the number of exosomal circRNA-related articles and their citations from 2015 to December 11, 2024. The first article on exosomal circRNAs was published in 2015, and the number of related publications consistently grew, with a particularly sharp rise occurring between 2019 and 2022. However, after reaching 177 publications in 2022, the number began to decline, and it is expected to return to 2020 levels in 2025. On the other hand, while the number of citations has continued to rise, the growth rate noticeably slowed after 2022.

### Distribution of countries/regions and institutions

**Figure 3:**
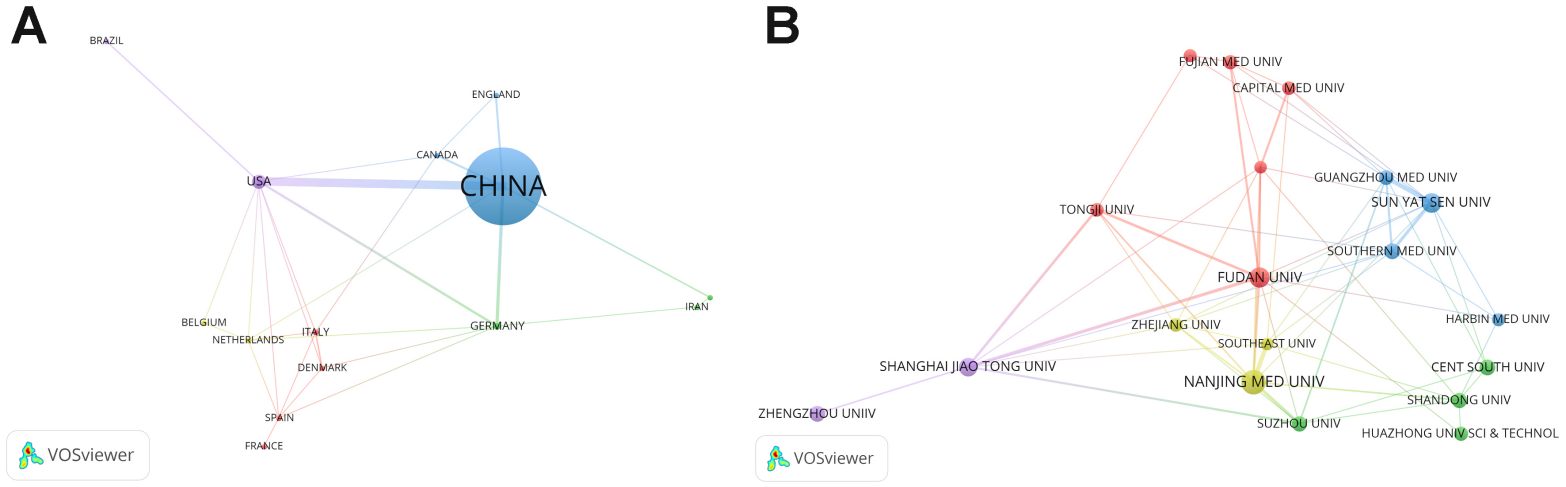
Collaboration in exosomal circRNA research, based on VOSviewer. (A) The co-authorship network among countries. The size of each node indicates the publication count, while the connecting lines between nodes indicate the strength of collaboration; (B) The co-authorship network among institutions.

A total of 32 countries/regions have participated in exosomal circRNA research. The top 10 countries/regions based on publication output are shown in Table 1. China ranks first with 708 articles and 23,142 citations, far surpassing the United States, which ranks second with 43 articles and 3,760 citations. These two countries also have the highest H-index values, 81 and 23, respectively. In total, 747 institutions contributed to exosomal circRNA research [Table 2]. In terms of output, Fudan University (61, 3.62%), Sun Yat-sen University (42, 2.50%), and Nanjing Medical University (40, 2.37%) rank at the top. The top 10 institutions are all from China, which aligns with China’s leading position in the number of published articles. Figure 3A illustrates the collaboration network for these countries/regions and institutions. The collaboration strength between China (39) and the United States (36) is the highest, followed by Germany (14), with other countries showing lower collaboration strength. Figure 3B shows that Fudan University is at the center of the network, with the strongest connection strength (26), followed by Sun Yat-sen University (21) and Nanjing Medical University (21), which form the core of their respective clusters.

**Table 1:** The Top 10 countries with the most published articles

**Rank**	**Country**	**Documents**	**Citations**	**Citations/documents**	**Total link strength**	**H-index**
1	China	708 (84.47%)	23,142	32.7147	39	81
2	United States	43 (5.13%)	3,760	87.4419	36	23
3	Germany	13 (1.55%)	769	59.1538	14	8
4	Italy	10 (1.20%)	522	52.2	6	9
5	Canada	5 (0.60%)	85	49.4	6	7
6	Denmark	5 (0.60%)	247	17	5	4
7	Iran	5 (0.60%)	23	4.6	2	6
8	Australia	4 (0.48%)	54	38.75	3	4
9	England	4 (0.48%)	155	13.5	4	6
10	Netherlands	4 (0.48%)	27	26	6	3

**Table 2:** The Top 10 institutions with the most published articles

**Rank**	**Institution**	**Documents**	**Citations**	**Citation/document**	**Total link strength**
1	Fudan University	61 (3.62%)	3,704	60.7213	26
2	Sun Yat-sen University	42 (2.50%)	3,060	72.8571	21
3	Nanjing Medical University	40 (2.37%)	1,640	41	21
4	Zhengzhou University	34 (2.02%)	1,364	40.1176	18
5	Shanghai Jiaotong University	26 (1.54%)	641	24.6538	4
6	Zhejiang University	26 (1.54%)	733	28.1923	2
7	Nantong University	25 (1.48%)	776	31.04	13
8	Southern Medical University	23 (1.36%)	1,102	47.913	8
9	Central South University	23 (1.36%)	676	29.3913	14
10	Huazhong University of Science and Technology	22 (1.30%)	1,363	61.9545	17

### Analysis of journals and co-cited journals

**Figure 4:**
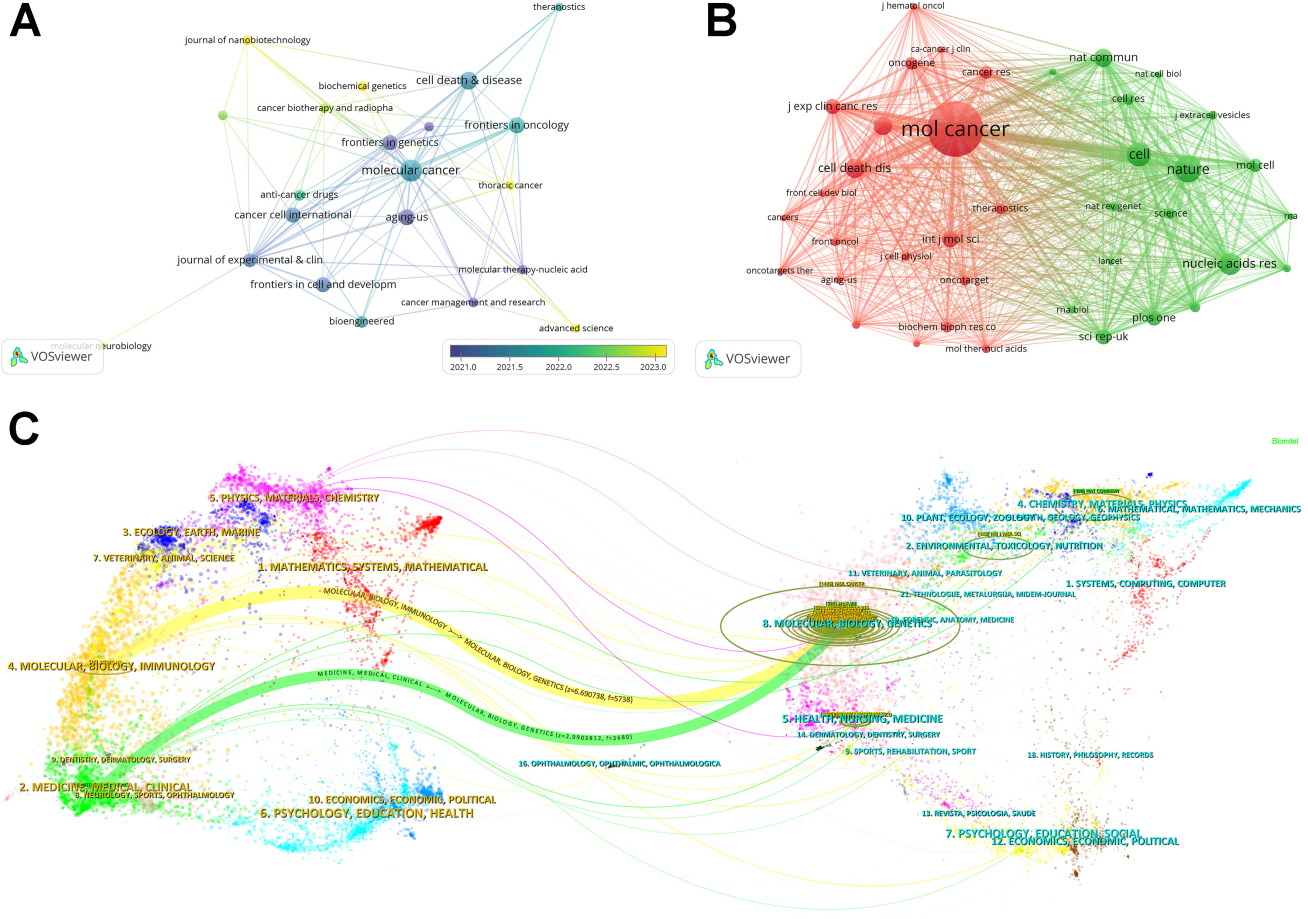
Journal distribution in exosomal circRNA research. (A) Journals publishing research on exosomal circRNAs, based on VOSviewer. The closer the label is to yellow, the newer the average publication year of the journal; (B) Distribution of exosomal circRNA research across various co-cited journals, based on VOSviewer; (C) The dual-map overlay of journals on exosomal circRNAs, based on CiteSpace. The citing journals on the left represent the frontier areas of exosomal circRNA research, while the cited journals on the right form the knowledge base of the field. Cited relationships are defined by the connecting lines, with the *f*-value indicating the citation counts.

The 767 publications on exosomal circRNAs were published across 332 journals. Using VOSviewer, we filtered and visualized 21 core journals with a publication threshold of 8 [Figure 4A]. Table 3 displays the top 10 journals according to publication volume. Ranking first with 21 publications, Molecular Cancer is the most influential journal in this area, boasting 2,023 citations. Other high-impact journals include Cell Death & Disease (17), Aging-US (15), and Frontiers in Oncology (15). The Impact Factor (IF) is an important measure of a journal’s influence, calculated by the average number of citations per article^[37]^. Among the top 10 journals, *Molecular Cancer* holds the highest IF2023 and the highest average citations per article, with values of 27.7 and 139, respectively. *Journal of Experimental and Clinical Cancer Research* (11.4, 106) and *Cell Death and Disease* (8.1, 60.1765) follow. These journals form the core platform for publishing research on exosomal circRNAs.

**Table 3:** The top 10 journals with the most published articles

**Rank**	**Journal**	**Document**	**Citation**	**Total link strength**	**IF2023**	**JCR**
1	*Molecular Cancer*	21	2,919	119	27.7	Q1
2	*Cell Death & Disease*	17	1,023	45	8.1	Q1
3	*Aging-US*	15	597	33	3.9	Q2
4	*Frontiers in Oncology*	15	230	50	3.5	Q2
5	*Cancer Cell International*	14	375	27	5.3	Q1
6	*Frontiers in Cell and Developmental Biology*	14	379	16	4.6	Q1
7	*Frontiers in Genetics*	14	455	37	2.8	Q2
8	*Journal of Experimental & Clinical Cancer Research*	13	1,378	75	11.4	Q1
9	*Bioengineered*	11	207	16	4.2	Q2
10	*Anti-Cancer Drugs*	10	105	15	1.8	Q3

A co-citation relationship is formed when two papers are cited together in the same article, which helps assess the impact of the cited articles or journals in a specific research area^[38]^. Using VOSviewer, we identified the co-citation network of 39 journals with a citation threshold of 167 [Figure 4B]. Detailed information is included in Table 4. *Molecular Cancer* leads with 1,426 citations, followed by *Nature* (705), *Cell* (627), and *Nucleic Acids Research* (573) in citation frequency. In the co-citation network, *Molecular Cancer* and *Nature* are the core nodes of their respective clusters. Of the top 10 co-cited journals, five have an IF2023 greater than 10, namely *Molecular Cancer* (27.7), *Nature* (50.5), *Cell* (45.6), *Nucleic Acids Research* (16.7), and *Nature Communications* (14.7).

**Table 4:** The top 10 co-cited journals

**Rank**	**Journal**	**Citations**	**Total link strength**	**IF2023**
1	*Mol Cancer*	1,426	24,604	27.7
2	*Nature*	705	15,066	50.5
3	*Cell*	627	15,860	45.6
4	*Nucleic Acids Res*	573	12,683	16.7
5	*Cell Death Dis*	502	10,670	8.1
6	*Nat Commun*	480	10,747	14.7
7	*Cancer Lett*	475	10,560	9.1
8	*Int J Mol Sci*	419	8,917	4.9
9	*PLOS One*	400	8,962	2.9
10	*Sci Rep-UK*	399	8,129	3.8

Further, we used CiteSpace for dual-map overlay analysis of journals, which reveals the association between journals and co-cited journals and shows the citation paths in the literature [Figure 4C]. Two main citation paths were identified: research published in MOLECULAR/BIOLOGY/GENETICS journals was mainly cited by MOLECULAR/BIOLOGY/IMMUNOLOGY (f = 5,738) and MEDICINE/MEDICAL/CLINICAL (f = 2,680) journals.

### Authors and co-cited authors

**Figure 5:**
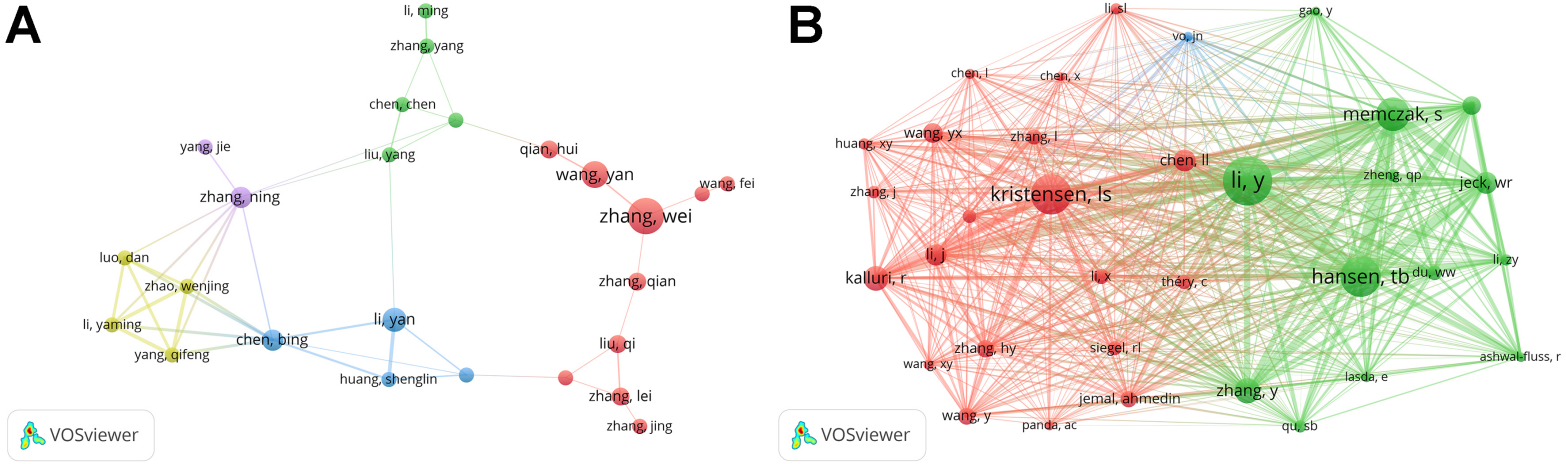
Author collaboration network in exosomal circRNA research, based on VOSviewer. (A) The co-authorship network among authors; (B) The network among co-cited authors.

A total of 5,200 authors and 16,894 co-cited authors have published articles related to exosomal circRNAs. We visualized the collaboration network of these authors using VOSviewer [Figure 5], and the top 10 authors contributing the most are shown in Tables 5 and 6. Zhang Wei (12), Wang Yan (9), Li Yan (8), and others have published the most articles. Chen Bing (82), Zhang Wei (54), and Qian Hui (41) are the top 3 authors with the highest H-index. Figure 5A displays the collaboration network among 38 authors, each having contributed at least 5 articles. These 38 authors are divided into 5 clusters, each forming a local collaborative network, with relatively few collaborations between different author groups. Among the co-cited authors, Li Y (267), Hansen TB (221), and Kristensen LS (221) are the most frequently cited [Figure 5B]. The H-index is used to evaluate a researcher’s academic contribution and influence, indicating that a scholar has at least H papers cited H times, which considers both the quantity and quality of their work^[39]^. Tables 5 and 6 show that Chen Bing and Kalluri R have the highest H-index among authors and co-cited authors, indicating their exceptional research quality.

**Table 5:** The top 10 authors with the most published articles

**Rank**	**Authors**	**Document**	**Citation**	**H-Index**	**Institution**
1	Zhang, Wei	12	376	54	Zunyi Medical University (China)
2	Wang, Yan	9	407	11	Zunyi Medical University (China)
3	Li, Yan	8	925	13	Fudan University (China)
4	Chen, Bing	7	675	82	Fudan University (China)
5	Zhang, Ning	7	235	8	Central South University (China)
6	Liu, Qi	6	336	28	Peking University (China)
7	Qian, Hui	6	77	41	Jiangsu University (China)
8	Zhang, Lei	6	83	32	Zhejiang University (China)
9	Zhang, Qian	6	214	33	Zunyi Medical University (China)
10	Chen, Chen	5	376	15	Zunyi Medical University (China)

**Table 6:** The top 10 co-authors with the most published articles

**Rank**	**Authors**	**Citations**	**Total link strength**	**H-Index**	**Institution**
1	Li, Y	267	1,382	13	Fudan University (China)
2	Hansen, TB	221	1,243	31	Aarhus University (Denmark)
3	Kristensen, LS	221	1,011	31	Aarhus University (Denmark)
4	Memczak, S	182	1,071	10	Max DelbrückCenter (Germany)
5	Zhang, Y	138	752	39	Nantong University (China)
6	Kalluri, R	134	615	124	Baylor College of Medicine (USA)
7	Chen, LL	120	581	80	ShanghaiTech University (China)
8	Jeck, WR	120	768	21	University of North Carolina (USA)
9	Li, J	118	635	77	Third Military Medical University (China)
10	Wang, YX	107	493	8	Zhengzhou University (China)

### Co-cited references and references citation burst

The foundation of a discipline’s knowledge is formed by co-cited references^[40]^. By analyzing co-cited references, we reveal the research results on which the study of exosomal circRNAs is based. Table 7 presents the 10 most cited co-references, five of which have accumulated over 100 citations: Li Yan (222), Hansen Thomas B (193), Memczak Sebastian (159), Kristensen Lasse S. (151), and Wang Yangxia (102). This result aligns with the co-cited author analysis, indicating that these articles are the cornerstone of the discipline. Notably, the most cited paper, titled “Circular RNA is enriched and stable in exosomes: a promising biomarker for cancer diagnosis”, was the first to reveal, through RNA sequencing, that exosomes in human blood circulation contain abundant and stable circRNAs. These circRNAs are widely and stably present in cancer cell-derived exosomes. Moreover, exosomal circRNAs in serum can effectively differentiate colorectal cancer patients from healthy controls^[41]^. This paper is the origin of research on exosomal circRNAs and has become the factual foundation for subsequent studies.

**Table 7:** The top 10 co-cited references based on the number of citations

**Rank**	**Citations**	**Title**	**First author**	**Document type**	**Year**
1	222	Circular RNA is enriched and stable in exosomes: a promising biomarker for cancer diagnosis	Li, Yan	Letter	2015
2	193	Natural RNA circles function as efficient microRNA sponges	Hansen, Thomas B	Article	2013
3	159	Circular RNAs are a large class of animal RNAs with regulatory potency	Memczak, Sebastian	Article	2013
4	151	The biogenesis, biology and characterization of circular RNAs	Kristensen, Lasse S	Review	2019
5	102	Exosomal circRNAs: biogenesis, effect and application in human diseases	Wang, Yangxia	Review	2019
6	92	The biology, function, and biomedical applications of exosomes	Kalluri, Raghu	Review	2020
7	86	Global cancer statistics	Jemal, Ahmedin	Article	2011
8	78	Circular RNAs are abundant, conserved, and associated with ALU repeats	Jeck, William R	Article	2013
9	68	Circular RNA profiling reveals an abundant circHIPK3 that regulates cell growth by sponging multiple miRNAs	Zheng, Qiupeng	Article	2016
10	54	circRNA Biogenesis Competes with Pre-mRNA Splicing	Ashwal-Fluss, Reut	Article	2014

**Figure 6:**
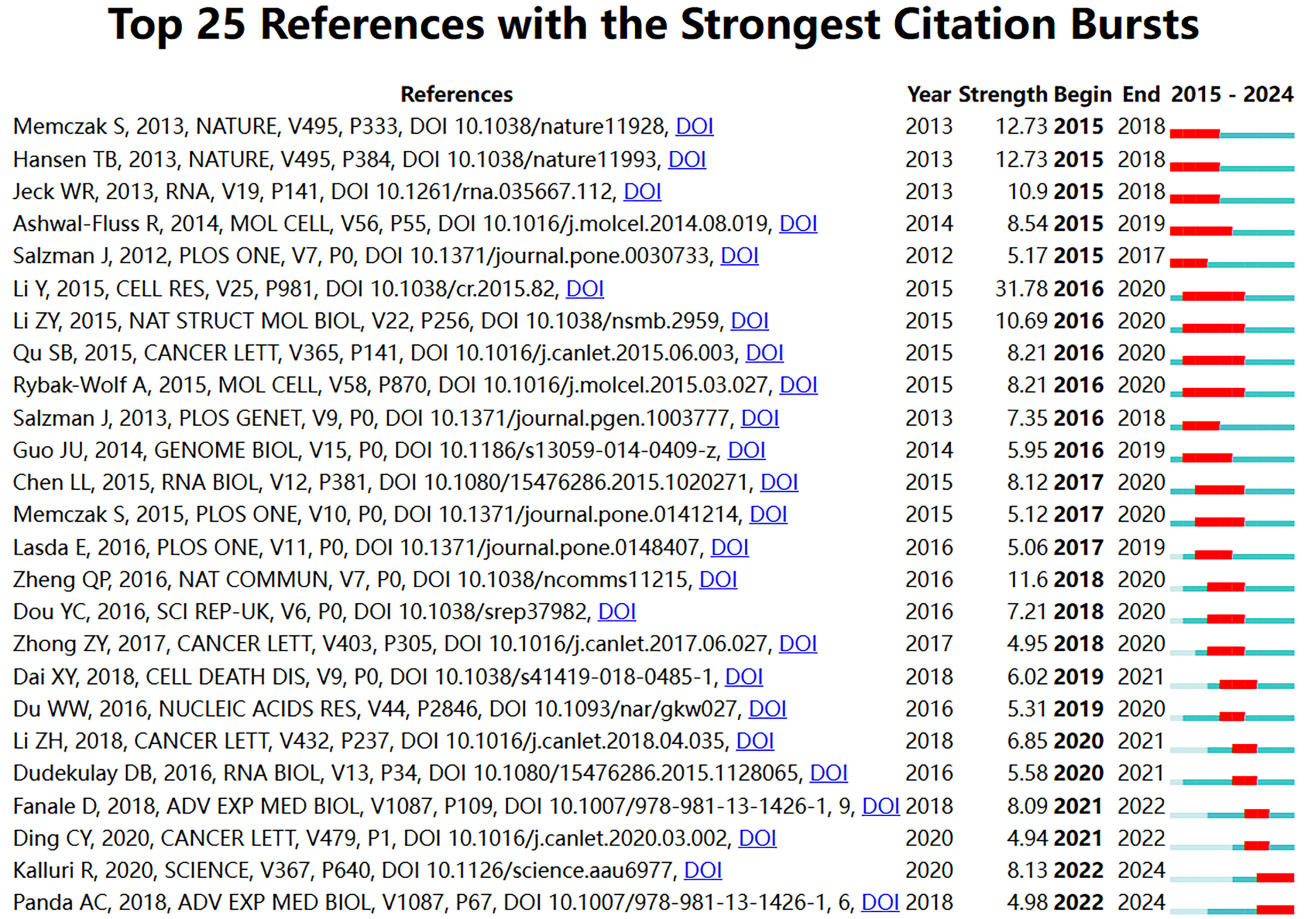
The top 25 references with the strongest citation bursts, based on CiteSpace. Citation burst reflects a sharp increase in citation frequency. The red bars indicate the start and end time intervals of the burst.

Furthermore, by analyzing reference bursts, we identified the top 25 articles with the most significant citation bursts. Citation bursts refer to a phenomenon where the citation frequency of a particular article sharply increases over a certain period, which can help predict research frontiers^[40]^. Figure 6 displays the top 25 references with the strongest citation bursts. The strongest burst is for Li Y’s Circular RNA is enriched and stable in exosomes: a promising biomarker for cancer diagnosis. Recent bursts occurred from 2022 onwards, including Kalluri R’s The biology, function, and biomedical applications of exosomes, and Panda AC’s Circular RNAs act as miRNA sponges. Additionally, we used the bibliometrix package to obtain the top 10 locally cited papers. “Locally cited” refers to the results produced within a discipline being cited by the same discipline. The details are provided in [Supplementary Table 1].

### Keywords and hotspots

**Figure 7:**
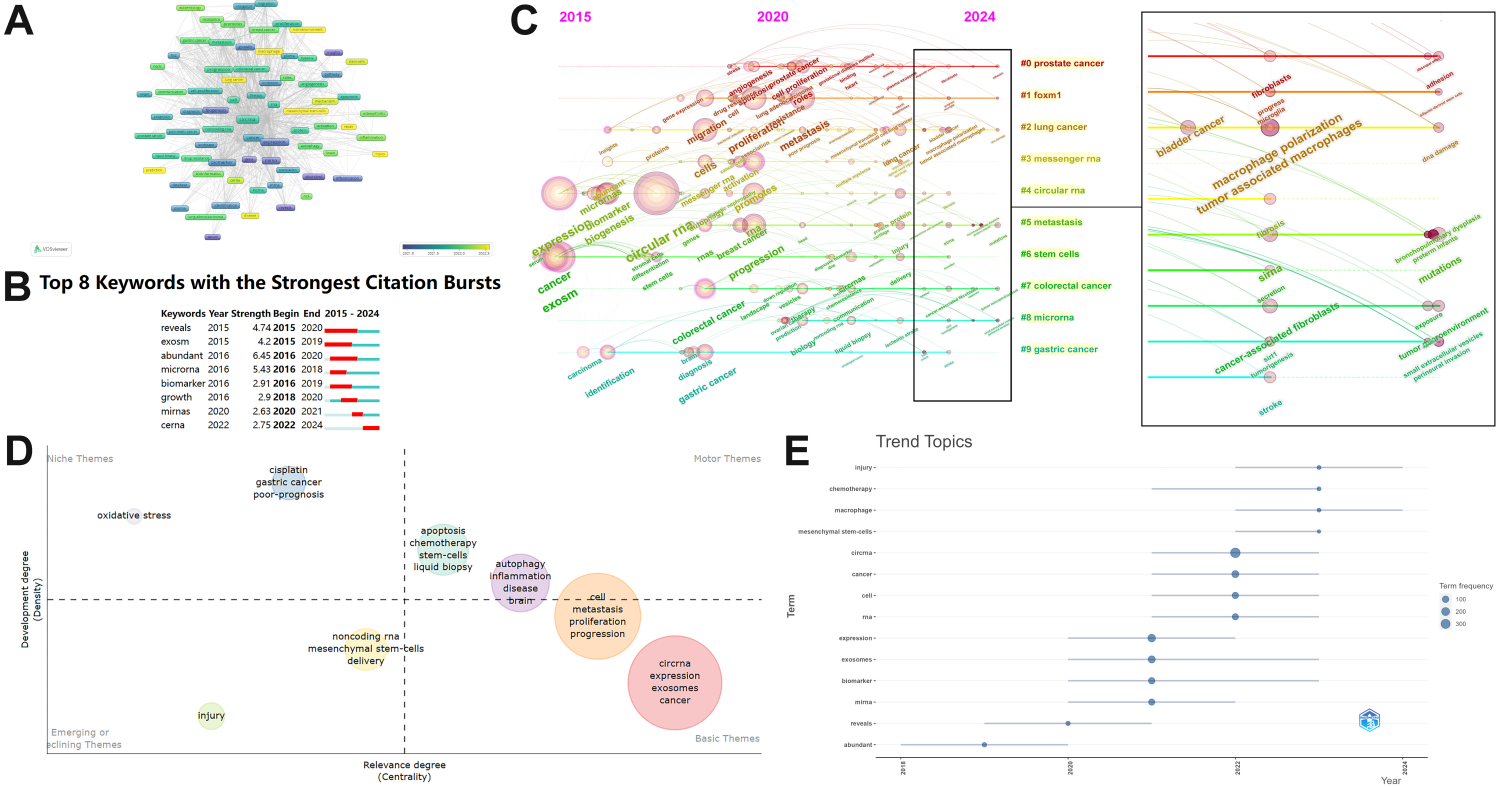
Research hotspots and trends in exosomal circRNAs. (A) Network of keywords with the most recent average publication year, based on VOSviewer; (B) The top 12 keywords with the strongest citation bursts, based on CiteSpace; (C) Time-zone viewer map related to exosomal circRNAs, based on CiteSpace; (D) Thematic map related to exosomal circRNAs, based on R; (E) Trend topics related to exosomal circRNAs, based on R.

Keywords are highly concise summaries of the themes of the literature. By analyzing keywords, we can understand the hotspots in the research field. We collected 2,510 keywords using VOSviewer, and Table 8 presents information on the top 30 keywords, among which “circRNA”, “exosomes”, “expression”, “biomarker”, “cancer”, “miRNA”, and “extracellular vesicle” appeared more than 100 times, which are likely the most mentioned topics in exosomal circRNA research. We visualized 76 keywords with co-occurrence frequency greater than 9, and in Figure 7A, keywords closer to yellow represent newer terms on average. Table 9 shows these recent keywords, categorized by clusters, including “macrophage” and “microenvironment.” The software considers keywords within the same cluster to have a higher similarity or relevance. Additionally, we also analyzed keyword citation bursts [Figure 7B], with the latest keywords being “ceRNA” and “tumor-associated macrophages.”

**Table 8:** The top 30 keywords with the highest occurrence frequency

**Rank**	**Keywords**	**Count**	**Rank**	**Keywords**	**Count**
1	circRNA	482	16	promotes	57
2	Exosomes	462	17	Colorectal cancer	49
3	Expression	175	18	Migration	49
4	Biomarker	149	19	HCC	48
5	Cancer	131	20	Gastric cancer	41
6	miRNA	110	21	ceRNA	39
7	Extracellular vesicle	107	22	Angiogenesis	38
8	Cell	93	23	Mechanism	38
9	Metastasis	91	24	Diagnosis	36
10	RNA	90	25	mRNA	35
11	Proliferation	87	26	Apoptosis	33
12	Progression	84	27	Breast cancer	33
13	Growth	65	28	Identification	33
14	Biogenesis	64	29	Protein	32
15	Invasion	60	30	Roles	31

**Table 9:** The keywords with the most recent average publication year

**Cluster**	**Keywords**	**Avg. pub. year**	**Count**	**Cluster**	**Keywords**	**Avg. pub. year**	**Count**
1	Macrophage	2022.8889	27	3	ceRNA	2022.2821	39
1	Microenvironment	2022.6364	11	3	Bioinformatics	2022.08	25
1	Cisplatin	2022.1818	22	3	Communication	2022	10
1	Epidemiology	2022.1667	18	4	Prediction	2022.5	10
2	Repair	2022.5455	11	4	Prostate cancer	2021.8235	17
2	Injury	2022.5333	15	4	Liquid biopsy	2021.8	15
2	Mesenchymal stem cells	2022.5	12	4	Noncoding RNA	2021.7778	27
2	Mechanism	2022.3684	38	5	Lung cancer	2022.5	18
3	Disease	2022.4375	16	5	Nsclc	2022.037	27

The distribution of keywords over time is depicted in the time-zone map, contributing to the understanding of how research hotspots evolve [Figure 7C]. In the time-zone map, keywords like “circRNA” and “exosome” reflecting the subject matter of the field appeared first. As time progressed, research began to focus on various cancers and directions such as “proliferation”, “metastasis”, “angiogenesis”, and “resistance.” After 2021, notable keywords such as “tumor microenvironment”, “cancer-associated fibroblasts”, “mesenchymal stem cells”, and “tumor-associated macrophages” emerged. These changes indicate that researchers’ interests are gradually shifting toward the TME.

To further explore the dynamic changes in research hotspots, we utilized the bibliometrix package to generate thematic maps and trend topics. As shown in Figure 7D, topics with higher centrality indices are considered core to the research field, while those with higher density indices represent more mature themes. The themes in the motor themes quadrant, such as autophagy and apoptosis, have developed well and are significant to the field of study. Topics in the basic themes quadrant, such as “circRNAs”, “cell”, and “metastasis”, represent foundational concepts in the research area. Although their development is still incomplete, they are crucial. The themes in the emerging or declining themes quadrant, such as “noncoding RNA”, “mesenchymal stem cells”, and “injury”, may either be newly emerging or nearing obsolescence. The themes in the niche themes quadrant, like “cisplatin” and “oxidative stress”, are more specialized and have limited relevance to the broader research field. In the trend topics [Figure 7E], it is evident that “injury” and “macrophages” are the most recent research topics along the timeline.

## DISCUSSION

### General information

This study involved a bibliometric analysis of 767 articles published between 2015 and December 11, 2024, focusing on exosomal circRNAs. The research on exosomal circRNAs began in 2015 when Li *et al*. first confirmed the widespread and stable presence of circRNAs in circulating exosomes, laying the foundation for subsequent studies^[41]^. As depicted in Figure 2, the publication count increased rapidly starting in 2018, possibly due to the establishment of the exoRBase database, which allowed researchers to more conveniently and comprehensively access circRNAs data^[42]^. Additionally, most landmark research results were published around 2020 [Supplementary Table 1], which contributed to a greater number of scholars becoming involved in this field. However, starting in 2022, the number of publications per year seems to be declining. Although this does not necessarily indicate that the research boom in exosomal circRNAs is waning, the absence of new groundbreaking findings may slow the pace of development in this field over the next few years.

In the field of exosomal circRNAs, China has published the most, representing 84.47% of all publications. Previous bibliometric studies have shown that Chinese scholars exhibit a stronger interest in ncRNAs compared to scholars from other countries^[43]^. Among the milestone achievements identified by bibliometrix [Supplementary Table 1], a large portion of contributions from China were funded by the National Natural Science Foundation of China (NSFC), suggesting that national policy encouragement may have played a certain role in driving this progress. However, the average citation count per article for China is only 32.7147, ranking 8th among all countries. Interestingly, China’s H-index (81) remains significantly higher than that of other countries. This suggests that China has a substantial amount of high-quality research in this field. Still, the large number of publications also includes many lower-impact studies, which brings down the average citation count. Chinese researchers should continue to maintain their advantage in the number of publications while also focusing on improving research quality, which will have a more positive impact on exosomal circRNAs studies. The majority of active institutions are located in China, with Fudan University, Sun Yat-sen University, and Nanjing Medical University being the most prominent. In terms of collaboration, China and the United States have a strong collaborative relationship, but cooperation with other countries is limited, with some countries even having no collaboration at all. While there are collaboration networks between different institutions, most are confined to domestic exchanges. We recommend that countries and institutions eliminate research barriers, strengthen academic exchanges, and promote the development of exosomal circRNA research.

A considerable number of journals are willing to publish research on exosomal circRNAs. Among the top ten journals by publication count, half are from the JCR Q1 zone. However, only 10 out of the 332 journals have more than 10 articles on this topic. On the one hand, the research on exosomal circRNAs is relatively limited; on the other hand, high-impact journals are more inclined to accept high-quality research. Most of the 767 articles on exosomal circRNAs have been published in journals with relatively low IF, which indirectly suggests that the quality of the current research on exosomal circRNAs still has room for improvement. *Molecular Cancer* is the most active journal, publishing the most studies and being cited the most by research on exosomal circRNAs. These studies primarily focus on the molecular mechanisms of exosomal circRNAs in various cancers. Considering both the acceptance rate and IF2023, we recommend that researchers submit their work to these journals [Table 3]. In Figure 4C, the dual-map overlay of journals illustrates the current distribution of journal topics. At present, research on exosomal circRNAs has begun to shift toward clinical applications, with an increasing focus on immune-related issues.

According to the H-index, Zhang Wei is ranked as the top author in terms of productivity and influence. Following closely is Wang Yan, who is from the same research team that previously discovered the association between M2 macrophage-derived exosomal circRNAs and myocardial fibrosis after a heart attack^[44]^. The team’s latest study indicates that the system for delivering exosome-encapsulated circRNA mSCAR preferentially targets mitochondria within macrophages, promoting macrophages to polarize toward the M2 phenotype^[45]^. These findings suggest a connection between exosomal circRNAs and M2 macrophages. The most cited co-cited author is Li Yan, whose team first confirmed the widespread and stable presence of circRNAs in exosomes^[41]^. Hansen TB, Kristensen LS, and Memczak S are pioneers in the field of circRNAs. They were the first to summarize the biological functions of circRNAs and to propose the groundbreaking hypothesis of circRNAs acting as miRNA sponges, which researchers should revisit and learn from^[12,20,46]^. Additionally, Kalluri R. is the most cited author with the highest H-index in the field of exosomal circRNAs, exerting a significant influence. His team has recently focused on studies related to the TME^[47,48]^. Scholars studying the relationship between exosomal circRNAs and the TME should consider his work.

### The hotspots and frontiers

A time-zone map can intuitively show the changes in hotspots over different years. Figure 7C shows that the number of co-occurring keywords each year corresponds with the annual number of publications. From 2015 to 2017, the number of publications per year was fewer than 10, resulting in a lower number of co-occurring keywords, which mostly reflected disciplinary research themes such as “circular RNA”, “cancer”, and “exosome.” This also explains why these were identified as basic themes, representing fundamental concepts in the research field. Most of the research in this phase was centered around the expression of circRNAs in body fluids in different pathological and physiological states^[49,50]^. Starting in 2018, the number of keywords significantly increased. Emerging keywords included various cancers such as “colorectal cancer”, “gastric cancer”, and “breast cancer”, as well as terms like “pathway”, “activation”, and “angiogenesis.” Researchers paid particular attention to tumor biology, discovering the functions of exosomal circRNAs in different cancers^[51,52]^. In addition, keywords such as “biomarker”, “resistant”, “diagnosis”, and “therapy” suggest that the clinical value of exosomal circRNAs has gradually gained more attention. For instance, hsa_circ_0000419 was found to potentially serve as a novel gastric cancer screening marker, and exosomal circATG4B was shown to induce chemotherapy resistance in colorectal cancer^[53,54]^. After 2021, due to a decrease in publications and limited accumulation of data, the number of emerging keywords and co-occurrence frequency declined in the past two years. This phase saw the appearance of similar keywords like “tumor-associated macrophages”, “tumor microenvironment”, and “cancer-associated macrophages”, possibly influenced by the growing academic interest in TME concepts^[55]^. These trends in research changes were also reflected in the trend topics [Figure 7E], where the basic themes like “reveals” and “miRNA” gradually transitioned to TME-related terms like “macrophage” and “mesenchymal stem cells.”

As early as 2015, researchers began focusing on the direction of cancer research. As many biological functions of exosomal circRNAs in cancer remain unexplored, research after 2018 primarily concentrated on uncovering the molecular mechanisms through which exosomal circRNAs affect cancer^[56,57]^. However, most studies limited their exploration of molecular mechanisms to single-cell-level verifications and heavily relied on *in vitro* models, overlooking the complex microenvironment in tumors. Moreover, current limitations in exosome separation technologies, including extraction and analysis standardization, have hindered the development of exosomal circRNA research^[58]^. The lack of basic research further prevents the large-scale clinical translation of findings related to exosomal circRNAs. While researchers have recognized the clinical value of exosomal circRNAs, the discussion often remains speculative, particularly regarding their roles as biomarkers. This explains why “biomarker” is one of the most frequent and earliest keywords. Exosomal circRNAs’ role in inducing chemotherapy resistance is another frequently discussed topic. The bibliometric analysis identified chemotherapy as a well-developed theme and the latest trending topic [Figures 7D and E], with resistance emerging as the fourth-largest keyword in 2019 [Figure 7C]. Due to their crucial role in drug resistance development, exosomal circRNAs have even been referred to as the “chief culprit” in chemotherapy resistance^[59]^. However, most of the above findings remain at the laboratory stage, with discussions on their clinical value, such as therapeutic targets and diagnostic standards, being mostly speculative. Large-scale clinical trials to confirm their reliability are still lacking. As of January 2025, there are fewer than 10 clinical trials related to circRNAs, and fewer than 50% of these trials focus on exosomal circRNAs, with most experiments still in Phase 0 [Supplementary Table 2] (data from https://www.chictr.org.cn/ and https://clinicaltrials.gov/). In summary, research on exosomal circRNAs needs more clinical trials to strengthen clinical translation, while further improvement in basic research is required. Therefore, it is necessary to proactively identify future research trends for subsequent studies.

To explore research trends, we cross-verified the results of references and keyword analysis. Among the latest keywords identified by VOSviewer were “microenvironment”, “macrophage”, and “mesenchymal stem cells” [Table 9], especially “microenvironment” and “macrophage”, which appear to be highly correlated and belong to the same cluster. From the time-zone map starting in 2021, “microenvironment”, “tumor-associated macrophages”, “mesenchymal stem cells”, and “fibroblasts” have been among the most significant keywords each year [Figure 7C]. In addition, the Bibliometrix package recognized “mesenchymal stem cells” as an emerging theme [Figure 7D] and identified “macrophage” as the latest trend topic [Figure 7E]. We reviewed the top 10 locally cited papers collected by the bibliometrix package [Supplementary Table 1]. Local citation recognition is a unique feature of the Bibliometrix package, indicating how research results within a specific field are cited by other studies within the same field, reflecting the impact of that research. The majority of these articles focus on tumors and explore the link between exosomal circRNAs and angiogenesis or tumor progression^[14,60]^. Ultimately, we believe that the TME may be a new research trend for ongoing research into exosomal circRNAs.

The TME consists of tumor cells, immune cells, tumor-associated endothelial cells, cancer-associated fibroblasts (CAFs), extracellular matrix (ECM), and other components^[55]^. It provides a favorable environment for tumor cell proliferation, promoting metastasis and infiltration. It is also tightly connected to immune escape in tumors and drug resistance^[61]^. In the TME, exosomes act as key mediators of communication between cells. By transporting abundant cargo molecules, they facilitate communication between different cells^[62,63]^. Among the cargo molecules encapsulated in exosomes, exosomal circRNAs significantly influence various aspects of tumor progression, such as proliferation, angiogenesis, and drug resistance^[64]^.

#### Tumor-associated macrophages (TAMs)

In the time-zone map, the top keyword in 2023 was “tumor-associated macrophages”, followed closely by macrophage polarization [Figure 7C]. TAMs are macrophages that infiltrate the tumor tissue or microenvironment, recruited and polarized by the TME, with the M2 phenotype TAMs being considered to promote tumor growth^[65,66]^. Tumor cell-derived exosomal circRNAs regulate M2 polarization. These circRNAs, delivered to macrophages, act as miRNA sponges, activating specific signaling pathways like the JAK/STAT pathway to induce M2 polarization^[67-69]^. Subsequently, M2 TAMs can release exosomal circRNAs that affect tumor cells. For example, in endometrial cancer, exosomal hsa_circ_0001610 derived from M2 macrophages reduces the sensitivity of endometrial cancer cells to radiotherapy^[70]^. A recent study found that using the HIF-1 alpha inhibitor PX-478 can suppress cancer cells from secreting exosomal circ-0100519, ultimately inhibiting M2 polarization^[71]^. Therefore, targeting specific points in the M2 polarization process to treat cancer is an important future research direction. Additionally, some exosomal circRNAs can inhibit M2 polarization, such as exosomal hsa_circ_0017252, which slows gastric cancer progression by inhibiting M2 polarization^[72]^. Packaging these therapeutic circRNAs into exosomes for delivery to the TAMs is a promising therapeutic strategy, but preventing off-target effects and optimizing the purification of exosomes and circRNAs remain current challenges^[73]^.

#### Mesenchymal stem cells (MSCs)

VOSviewer identified MSCs as an important keyword around 2022 and clustered them with keywords such as injury and repair [Table 9]. This is because MSCs are stem cells with multi-directional differentiation potential, capable of differentiating into various mesenchymal tissues for injury repair, and thus, many studies focus on their repair functions^[74]^. Exosomes derived from MSCs exert similar effects as MSCs themselves, with ncRNAs being important effector molecules. Based on this characteristic, some studies suggest that exosome-mediated circRNA delivery from MSCs can repair liver fibrosis, thus preventing liver cancer^[75]^. Compared to MSC transplantation, using MSC-derived exosomes for repair results in fewer side effects and has better clinical potential. However, its therapeutic effect and safety still lack further validation in animal models, and exosome extraction and preservation remain ongoing challenges^[76]^. Additionally, MSCs outside the tumor have homing abilities, meaning they can be recruited to tumor tissue and differentiate into tumor-associated mesenchymal stem cells (TA-MSCs), affecting tumor occurrence, invasion, and metastasis through exosomal circRNAs^[77-79]^. Although the mechanism by which MSCs are recruited and become part of the TME has been linked to exosomes, whether exosomal circRNAs participate in this recruitment process still requires more verification^[80,81]^. Further exploration of the molecular mechanisms will help find new therapeutic strategies.

#### CAFs

In the TME, CAFs secrete more than 50% of the ECM in solid tumors, producing various factors that promote tumor proliferation and vascularization and are linked to resistance and immune suppression^[82]^. Exosomal circRNAs heavily influence this process^[83,84]^. Since CAFs are the largest producers of solid tumor ECM and secreted factors, they significantly affect tumor progression, and tumor drug resistance is largely influenced by them^[85]^. For example, exosomal circZFR derived from CAFs can inhibit the STAT3/NF-kappa B pathway in liver cancer cells, promoting cisplatin resistance^[86]^. However, benefiting from CAFs’ comprehensive influence on tumor progression, their secreted exosomes are considered excellent carriers for therapeutic molecules^[85]^. In the latest study, researchers used tumor vascular-targeting peptides to modify CAF-derived exosomes, delivering circ_0004872 to osteosarcoma cells, and reversing drug resistance by encoding peptides^[87]^. This research shows that CAF-derived exosomal circRNAs have cancer therapeutic potential, and the peptide-coding ability of circRNAs is also worth investigating. Although CAFs’ therapeutic potential has been widely confirmed, research specifically focusing on CAF-derived exosomal circRNAs remains limited. On the one hand, the overall number of studies on CAF-secreted exosomal circRNAs is still small. On the other hand, due to their heterogeneity, different CAF subpopulations exert distinct effects on tumors^[88]^. Therefore, further exploration is needed to elucidate the molecular mechanisms through which CAF-derived exosomal circRNAs induce drug resistance. Additionally, single-cell sequencing could be used to identify differences in both the levels and functions of exosomal circRNAs produced by different CAF subgroups, which may help identify more clinically relevant therapeutic targets.

In summary, we believe that the TME represents a promising new direction for ongoing research on exosomal circRNAs. This concept offers researchers a novel pathway to understanding tumors, enabling a more comprehensive grasp of the complex landscape of tumors and the construction of a more refined molecular mechanism framework. However, at present, studies on molecular mechanisms are mostly limited to unidirectional signaling pathways (e.g., circRNAs-miRNAs-mRNAs), without delving into the complex competitive regulatory networks within the recipient cells. More research tends to isolate the members of the TME to explore the connection between tumor cells and their surroundings. Yet, the TME is essentially a complex ecosystem with heterogeneity, where interwoven effects exist between its various components, and exosomal circRNAs serve as important mediators of communication. For example, CAFs can secrete exosomal circ_0084043 to induce the proliferation of endothelial cells within the TME^[89]^. Therefore, despite a foundational body of research, the heterogeneity of the TME and intercellular cross-talk have yet to be fully elucidated. In the keyword analysis results of this study, some terms may suggest new research approaches. Among them, the emerging keyword “ceRNA” stands out, while “machine learning” is a newly introduced keyword in 2022 [Figures 7B and C]. A previous study explored the regulatory network involving exosomal circRNAs from a ceRNA perspective, suggesting that ceRNA regulatory networks may play a significant role in the TME^[90]^. The rise of the ceRNA concept provides a fresh perspective on understanding the function of exosomal circRNAs in the TME, especially in understanding and constructing the complex interactions between various cell types within the TME. With advancements in bioinformatics, machine learning has become an emerging tool in exosomal circRNA research. Researchers have successfully identified circRNAs that could serve as pan-cancer biomarkers using machine learning^[91]^. These studies indicate that machine learning may prove to be a powerful tool for uncovering the complex regulatory networks within the TME. Furthermore, single-cell RNA sequencing technology, which offers certain advantages in revealing TME heterogeneity and intercellular cross-talk, has gained increasing attention in TME research^[92]^. Therefore, we suggest that researchers explore the use of these new technologies, along with concepts such as ceRNA and machine learning, to investigate how different cell types in the TME interact through exosomal circRNAs.

This study offers a comprehensive analysis of the research progress on exosomal circRNAs over the past decade. Previous bibliometric research on exosomes or circRNAs exists, but this is the first analysis that specifically targets exosomal circRNAs. Additionally, prior studies have primarily focused on bibliometric analyses of exosomes or circRNAs in the context of specific diseases. In contrast, this study emphasizes the overall discipline of exosomal circRNAs, exploring the structure of this field and its future development^[93,94]^. Of course, this study also has certain limitations. In terms of data collection, WoS does not cover all research outcomes, which may result in some omissions. However, due to digital object identifier (DOI) discrepancies across different databases, it is currently challenging to conduct multi-database joint analyses to reduce such omissions^[95]^. Only including English-language literature may also overlook important studies in other languages, leading to language bias. Furthermore, citation inflation could affect the objective assessment of a paper’s impact due to factors like self-citation and negative citations. Older publications may have higher citation counts, yet high citation frequency does not necessarily equate to high quality. Citation analysis struggles to differentiate between motivations for citing, potentially overestimating the academic contributions of certain studies. Database indexing delays are another limitation that cannot be ignored. There is usually a certain delay between a paper’s publication and its final indexing, so this study may not have included the most recent findings, leading to an incomplete evaluation of the latest research trends.

As research advances, cancer is increasingly recognized as a complex ecosystem, and the concept of the TME has gained growing attention. In fact, research on cancer in the field of exosomal circRNAs has developed rapidly in recent years^[21]^. As a result, the trend among researchers in the field has shifted toward focusing on the TME. The concept of the TME links tumor cells and surrounding tissues into a complex and unified system, with exosomal circRNAs serving as one of the critical mediators facilitating communication within this system. The intricate network formed by exosomal circRNAs originating from both tumor and non-tumor cells profoundly impacts the dynamics of the TME. Our research suggests that the TME may be a new direction for exosomal circRNA research. It emphasizes that researchers need to incorporate emerging technologies and concepts into their studies, offering corresponding recommendations. The rapid development of exosomal circRNA research has spanned only a few years. However, insufficient basic research has hindered its clinical translation, and this field remains in its early stages. The rise of TME research has marked the dawn of a new era in exosomal circRNA research, but more effort is still required from us.

## DECLARATIONS

### Availability of data and materials

In this study, the datasets used and analyzed were obtained from the Web of Science Core Collection (WoSCC). The raw data supporting the findings of this study are available in this article and its Supplementary Materials. Further data are available from the corresponding authors upon reasonable request.

### Copyright

© The Author(s) 2025.
