# Supplementary material for: Tumor microenvironment: new era in exosomal circRNA research - a bibliometric analysis
Source: Extracell Vesicles Circ Nucl Acids. 2025 May 19;6(2):245–66. doi: 10.20517/evcna.2024.102 (PMC12367469; doi:10.20517/evcna.2024.102)
Supplement: Supplementary file 1 [file evcna-6-2-245-SupplementaryMaterials.pdf]

Supplementary Materials

Tumor microenvironment: new era in exosomal circRNA research - a bibliometric analysis

Hai-Quan Wang[1,#], Lu Zhang[1,#], Ming-Jie Li[1,2], Rong-Quan He[3], Di-Yuan Qin[4], Bin Li[1], Jian-Di Li[1], Ke-Jun Wu[1], Shi-De Li[1,5], Han He[1,6], Zhen-Bo Feng[1], Gang Chen[1]

[1]Department of Pathology, The First Affiliated Hospital of Guangxi Medical University, Nanning 530021, Guangxi, China.

[2]Department of Pathology/Forensic Medicine, The First Affiliated Hospital of Guangxi Medical University, Nanning 530021, Guangxi, China.

[3]Department of Oncology, The First Affiliated Hospital of Guangxi Medical University, Nanning 530021, Guangxi, China.

[4]Department of Computer Science and Technology, School of Computer and Electronic Information, Guangxi University, Nanning 530004, Guangxi, China.

[5]Department of Information Management and Information Systems, School of Information and Management, Guangxi Medical University, Nanning 530021, Guangxi, China.

[6]Department of Clinical Medicine, School of Basic Medical Sciences, Guangxi Medical University, Nanning 530021, Guangxi, China.

[#]Authors contributed equally.

Correspondence to: Prof. Gang Chen, Prof. Zhen-Bo Feng, Department of Pathology, The First Affiliated Hospital of Guangxi Medical University, No. 6 Shuangyong Road, Nanning 530021, Guangxi, China. E-mail: chengang@gxmu.edu.cn; fengzhenbo_gxmu@163.com

Supplementary Table 1. The top 10 Local-cited references based on the number of citations

Rank	Title	Local Citations	Global Citations	First Author	Year
1	exoRBase: a database of circRNA, lncRNA and mRNA in human blood exosomes	50	401	Li, Shengli	2018
2	Exosomal circPACRGL promotes progression of colorectal cancer via the miR-142-3p/miR-506-3p- TGF-β1 axis	47	311	Shang, Anqan	2020
3	Circular RNA IARS (circ-IARS) secreted by pancreatic cancer cells and located within exosomes regulates endothelial monolayer permeability to promote tumor metastasis	42	290	Li, Jie	2018
4	Exosomal circRNA-100338 promotes hepatocellular carcinoma metastasis via enhancing invasiveness and angiogenesis	39	288	Huang, Xiuyan	2020
5	Exosomal circSHKBP1 promotes gastric cancer progression via regulating the miR-582-3p/HUR/VEGF axis and suppressing HSP90 degradation	37	279	Xie, Mengyan	2020
6	Exosome-delivered circRNA promotes glycolysis to induce chemoresistance through the miR-122-PKM2 axis in colorectal cancer	34	379	Wang, Xinyi	2020
7	Circular RNAs Co-Precipitate with Extracellular Vesicles: A Possible Mechanism for circRNA Clearance	32	318	Lasda, Erika	2016
8	Circular RNA circNRIP1 acts as a microRNA-149-5p sponge to promote gastric cancer progression via the AKT1/mTOR pathway	32	601	Zhang, Xing	2019
9	Exosomal circRNA_100284 from arsenite-transformed cells, via microRNA-217 regulation of EZH2, is involved in the malignant transformation of human hepatic cells by accelerating the cell cycle and promoting cell proliferation	27	146	Dai, Xiangyu	2018
10	Tumor-released exosomal circular RNA PDE8A promotes invasive growth via the miR-338/MACC1/MET pathway in pancreatic cancer	27	271	Li, Zhonghu	2018

Supplementary Table 2. Current clinical trials on circRNAs

Registration number	Date of registration	Target disease	Study type	Clinical application	Sample name	Study phase
ChiCTR2400093481	2024	Gastric cancer	Diagnostic test	Biomarkers for diagnosis	Plasma	0
ChiCTR2300078812	2023	thyroid cancer	Basic science	Biomarkers for diagnosis	Tissue	0
ChiCTR2300069863*	2023	Cholangiocarcinoma	Observational study	Biomarkers for diagnosis	Blood	0
ChiCTR1900024188*	2019	Prostate cancer	Diagnostic test	Biomarkers for diagnosis	Urine	0
ChiCTR1800018038*	2018	Pancreatic Cancer	Diagnostic test	Biomarkers for predicting prognosis	Blood	Diagnostic new technique clincal study
ChiCTR1800019529	2018	Prostate cancer	Diagnostic test	Biomarkers for predicting prognosis	Plasma	Diagnostic new technique clincal study

The clinical trials marked with * focus on exosomal circRNAs as the research subjects.
